# SURPASS‐CVOT and the Emerging Biology of Cardiometabolic Benefit

**DOI:** 10.1111/dom.71175

**Published:** 2026-07-30

**Authors:** Salvatore Corrao, Francesco Cosentino, Massimo Federici

**Affiliations:** ^1^ Department of Clinical Medicine, Unit of Internal Medicine National Relevance and High Specialization Hospital Trust ARNAS Civico Di Cristina Benfratelli Palermo Italy; ^2^ Department of Health Promotion Sciences, Maternal and Infant Care, Internal Medicine and Medical Specialties (PROMISE) University of Palermo Palermo Italy; ^3^ Department of Medicine, Solna Karolinska Institutet and Karolinska University Hospital Stockholm Sweden; ^4^ Department of Systems Medicine University of Rome Tor Vergata Rome Italy

**Keywords:** cardiometabolic vulnerability, dual incretin agonism, endpoint specialisation, MACE, SURPASS‐CVOT, tirzepatide

## 1 The Question Raised by SURPASS‐CVOT

In SURPASS‐CVOT, tirzepatide achieved non‐inferiority versus dulaglutide for the primary composite of cardiovascular death, myocardial infarction, and stroke (HR 0.92; 95.3% CI 0.83–1.01; *p* = 0.003 for non‐inferiority) [1]. The non‐inferiority margin of 1.05 was pre‐specified to preserve at least 50% of the established cardioprotective effect of dulaglutide demonstrated in REWIND [2]. This calibration has, however, been contested: a margin derived solely from REWIND's point estimate would imply a considerably stricter threshold of approximately 1.005, under which non‐inferiority would not have been met, underscoring that margin selection remains a matter of methodological judgement rather than settled convention [3]. Indirect comparison across trials also warrants caution, since dulaglutide—rather than placebo—served as the active comparator, and the SURPASS‐CVOT and REWIND populations differed in diabetes duration, cardiovascular disease prevalence and baseline insulin use, factors that bear on the constancy assumption underlying non‐inferiority inference; other incretin‐based agents, including semaglutide, have shown larger absolute MACE reductions against placebo, underscoring that comparator choice remains part of this interpretive picture. Within this conservative inferential framework, non‐inferiority should not be read as absence of harm but as preservation of cardiovascular efficacy against an active cardioprotective comparator. The trial was event‐driven and primarily powered for non‐inferiority; the marginal miss on superiority (*p* = 0.09) is the expected outcome when a biologically potent agent is tested against an already effective comparator with a tight margin.

Once cardioprotection is established, the relevant scientific question shifts. It is no longer whether tirzepatide preserves cardiovascular efficacy against a stringent active comparator—SURPASS‐CVOT establishes that, even though superiority for MACE‐3P itself was not demonstrated—but on which dimensions of cardiovascular vulnerability it acts most distinctively, and whether the endpoints used to capture its effects are aligned with its underlying biology.

A directionally coherent pattern emerges across multiple secondary domains. All‐cause mortality favoured tirzepatide (HR 0.84; 95% CI 0.75–0.94) [1], as did the MACE‐4 composite that included coronary revascularization (HR 0.88; 0.81–0.96) [1]. In the pre‐specified exploratory kidney analysis, the primary composite renal outcome was reduced by 23% with tirzepatide (HR 0.77; 0.68–0.88; *p* = 0.0002), with consistent effects across CKD risk strata and a between‐group difference in eGFR slope of 0.93 mL/min/1.73m^2^ per year in high‐risk CKD [4]. Because superiority for the primary MACE‐3P endpoint was not formally established, the pre‐specified hierarchical testing procedure was halted at that step; mortality, MACE‐4 and the kidney outcomes reported here are therefore properly classified as exploratory and hypothesis‐generating rather than confirmatory—a distinction developed further elsewhere [5]—within this inferential limit, their consistency across mortality, cardiorenal and metabolic domains remains a pattern worth highlighting rather than dismissing as incidental.

**FIGURE 1:**
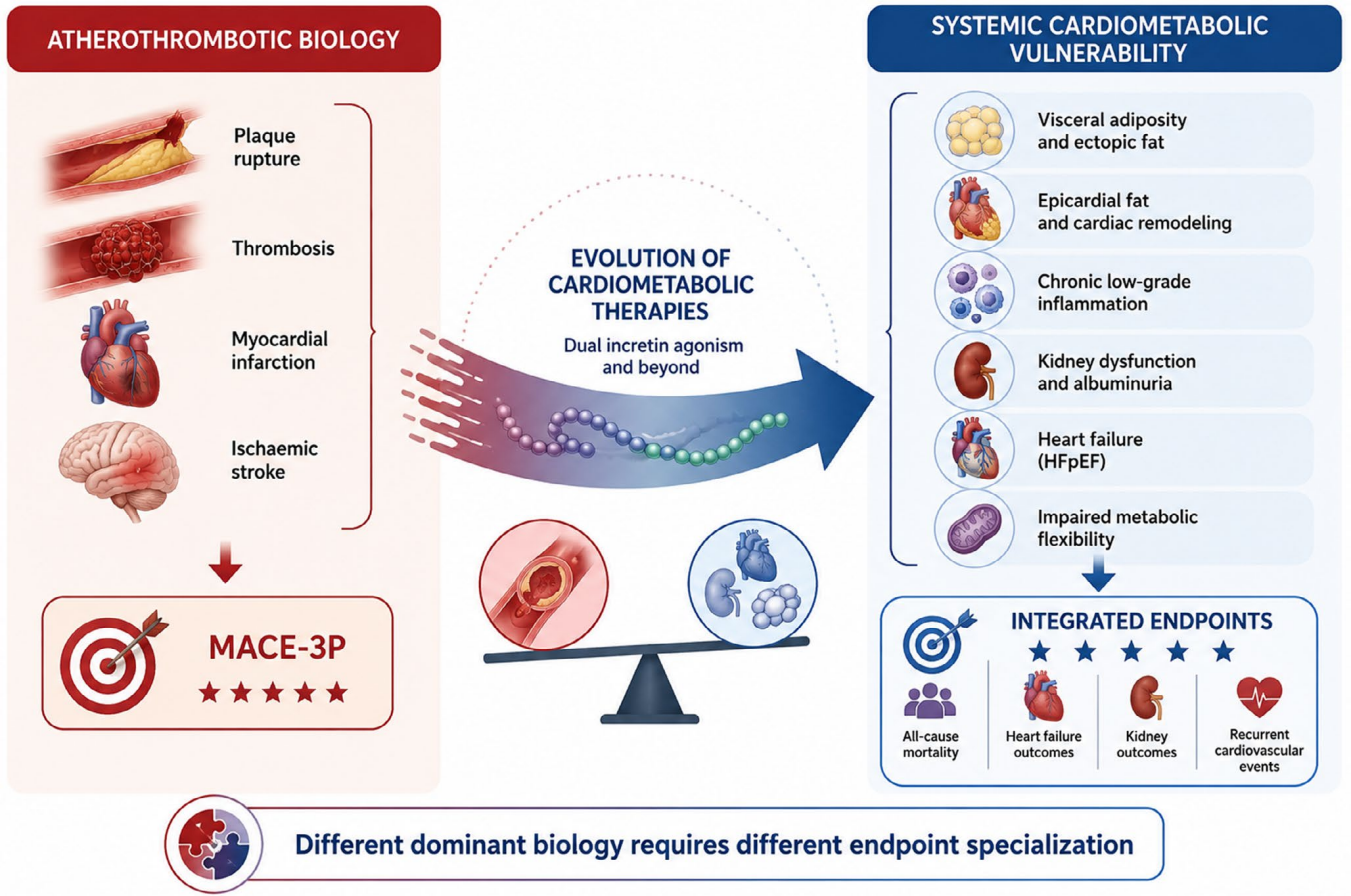
From atherothrombotic risk to systemic cardiometabolic vulnerability: Matching endpoints to therapeutic biology. The biological targets of cardiometabolic therapies have progressively expanded beyond plaque‐centred mechanisms towards systemic pathways linking adipose tissue dysfunction, inflammation, heart, kidney and metabolism. The figure is a conceptual, illustrative framework rather than a validated classification system: Individual therapies may act across overlapping biological domains simultaneously, and endpoint selection depends on the dominant expected treatment effect of the specific agent and population under study rather than on a strict binary categorisation. The figure proposes that cardiovascular outcome assessment should increasingly align endpoint architecture with the predominant biological domain targeted by therapy, providing a conceptual framework for interpreting trials of next‐generation cardiometabolic agents.

This directionally coherent pattern can be understood within a broader biological framework. As illustrated in Figure 1, the dominant biology targeted by a therapy may determine which cardiovascular endpoints most effectively capture its clinical benefit.

## 2 MACE‐3P and the Underlined Dominant Disease Biology

MACE‐3P is a precision instrument. Built to detect plaque rupture, thrombosis and recurrent ischaemic events, it has resolved the cardiovascular benefit of statins, antithrombotics and PCSK9 inhibitors with remarkable efficiency [6]. Its time‐to‐first‐event architecture privileges discrete atherothrombotic events as drivers of inference, and it remains the appropriate primary endpoint when atherothrombosis is the dominant disease biology.

However, cardiovascular risk in patients with type 2 diabetes, obesity and metabolic dysfunction is increasingly driven by mechanisms that lie beyond plaque biology: visceral and ectopic adiposity, immunometabolic inflammation, microvascular dysfunction, cardiorenal remodelling and the HFpEF phenotype that characterises the cardiovascular‐kidney‐metabolic (CKM) syndrome, a systemic disorder arising from pathophysiological interactions among metabolic risk factors, chronic kidney disease and cardiovascular disease, leading to multiorgan dysfunction and adverse cardiovascular outcomes [7]. These dimensions are not absent from MACE‐3P, but they are not its primary resolving domain. Therapies that act predominantly on these dimensions may produce signals that MACE‐3P captures incompletely—not because they lack cardiovascular efficacy, but because the endpoint is specialised for a different biological substrate.

## 3 Dual Incretin Agonism as Systemic Anti‐Vulnerability Therapy

Tirzepatide is not simply a sum of GIP and GLP‐1 agonism. It is a unimolecular co‐agonist that engages both incretin receptors—expressed across adipose tissue, skeletal muscle, myocardium, kidney and central nervous system—within a molecular network, generating a biological profile distinct from the additive combination of selective agonists [8]. This distinction matters because the cardiometabolic effects observed in SURPASS‐CVOT cannot be fully reduced to either receptor system in isolation.

A useful reframing concerns weight. Tirzepatide produces substantially greater body weight reduction than dulaglutide (between‐group difference −6.8 percentage points), but limiting the cardiometabolic effects as weight‐mediated obscures the relevant biology. The drug preferentially reduces visceral and ectopic adipose depots—epicardial, perivascular, renal sinus, intramyocellular and hepatic—which are biologically active drivers of organ dysfunction rather than passive markers of total adiposity [9]. A pooled mediation analysis of SURPASS trials estimated that only 45.9% of the improvement in albuminuria was indirectly mediated by HbA^1c^ and body weight [10], leaving more than half of the renal effect attributable to mechanisms operating beyond classical metabolic intermediates—a post hoc mediation finding rather than a mechanistic demonstration. Candidate mechanisms, not directly tested within SURPASS‐CVOT itself, plausibly include attenuation of epicardial adipose inflammation, reduction of immunometabolic signalling through IL‐6/TNF/NLRP3 pathways, improvement of microvascular function and restoration of metabolic flexibility under haemodynamic stress—hypotheses grounded in preclinical and observational evidence [11].

This biology converges on a single concept: systemic cardiometabolic vulnerability. Visceral and ectopic adiposity drive paracrine inflammation in adjacent organs, including the myocardium and the renal interstitium. Immunometabolic activation amplifies endothelial dysfunction and impairs cardiorenal coupling. Lipotoxicity and impaired metabolic flexibility predispose to congestion, the HFpEF phenotype and frailty [12, 13]. These dimensions are interconnected rather than sequential, and they are all targets of dual incretin biology. The therapeutic effect is not best described as ‘fewer plaque ruptures’, but as greater systemic physiological reserve under metabolic and haemodynamic stress.

If a therapy preferentially attenuates congestion biology, renal decline, ectopic adiposity and metabolic inflammation, the magnitude of mortality reduction may exceed the magnitude of MACE reduction—precisely because mortality and composite cardiorenal endpoints aggregate effects across the biological dimensions where the therapy acts most strongly, while MACE‐3P resolves a narrower atherothrombotic domain.

## 4 Implications for Cardiovascular Outcome Trials

The question is not whether MACE‐3P remains valid, but whether MACE‐3P alone remains sufficient when the therapeutic target is no longer atherothrombosis. The answer is increasingly that different therapies require complementary—rather than corrective—endpoint architectures alongside MACE‐3P, which retains unmatched resolving power for atherothrombotic risk.

The SGLT2 inhibitor experience offers a precedent. EMPA‐REG OUTCOME demonstrated cardiovascular benefit dominated not by recurrent myocardial infarction but by cardiovascular mortality and heart failure hospitalisation [14], a discordance that subsequently reshaped the endpoint architecture of cardiorenal trials including EMPEROR, DAPA‐HF and FLOW [15]. Tirzepatide may mark a comparable inflection for cardiometabolic therapies acting across multiple biological dimensions simultaneously. Lessons from STEP‐HFpEF and the developing evidence on incretin‐based therapies in obesity‐related HFpEF point in the same direction [16]. This logic is not unique to dual incretin agonism: the same reasoning increasingly applies across the expanding cardiovascular‐kidney‐metabolic (CKM) therapeutic class, including SGLT2 inhibitors and GLP‐1 receptor agonists, whose dominant biological actions likewise extend beyond atherothrombosis. Endpoint specialisation is therefore best understood as a class‐wide feature of modern cardiometabolic drug development rather than a property of any single molecule.

For future cardiovascular outcome trials of cardiometabolic agents, several design implications follow. Hierarchical composite endpoints incorporating all‐cause mortality, heart failure events and kidney progression may better capture the integrated biology than time‐to‐first‐event MACE. Recurrent‐event analyses and multistate models can detect benefits that classical time‐to‐first‐event approaches underestimate when therapies reduce systemic vulnerability without sharply altering the incidence of a single dominant atherothrombotic event. Precision‐oriented enrichment strategies—recruiting populations in whom metabolic stress and cardiorenal disease dominate risk—may further clarify where preserved cardiovascular biology translates into incremental clinical benefit. As multiagonist platforms incorporating glucagon and amylin receptor activity extend incretin‐based therapy across the broader cardio‐kidney‐liver‐metabolic (CKLM) spectrum, these endpoint considerations are likely to become more, not less, relevant.

A regulatory dimension deserves explicit consideration. The current framework, in which MACE‐3P remains the principal endpoint for cardiovascular safety and efficacy of glucose‐lowering and obesity therapies, was designed in an era of predominantly atherothrombotic risk. As the therapeutic landscape shifts towards agents acting on systemic cardiometabolic biology, regulatory expectations for cardiovascular endpoint architectures may need to evolve in parallel—not by abandoning MACE, but by recognising that endpoint specialisation is the appropriate response to therapeutic specialisation. The goal is not to replace one paradigm with another, but to match the resolving power of the endpoint to the dominant biology of the therapy under evaluation.

## 5 Coda

SURPASS‐CVOT does not invite a verdict on tirzepatide; that verdict is favourable—cardiovascular protection was established within the trial's pre‐specified non‐inferiority framework, while superiority for MACE‐3P narrowly missed statistical significance—against one of the most stringent active comparators yet deployed in this field. It invites a verdict on how we evaluate cardiometabolic therapies. The directionally coherent, if formally exploratory, pattern of effects observed across mortality, cardiorenal, and metabolic outcomes is consistent with—though not proof of—a therapy that reduces systemic cardiometabolic vulnerability rather than atherothrombosis alone. SURPASS‐CVOT suggests that the next frontier in cardiovascular outcome trials may no longer be the search for more effective therapies alone, but the development of endpoint architectures whose resolving power matches the biology of those therapies.

## Author Contributions

**Salvatore Corrao:** conceptualization, methodology, investigation, writing – original draft, writing – review and editing, and supervision. **Francesco Cosentino and Massimo Federici:** conceptualization, validation, and writing – review and editing. All authors contributed to the interpretation of the evidence, critically revised the manuscript for important intellectual content, approved the final version of the manuscript, and agree to be accountable for all aspects of the work.

## Funding

The authors have nothing to report.

## Ethics Statement

The authors have nothing to report.

## Conflicts of Interest

The authors declare no conflicts of interest.

## Data Availability

No new data were generated or analysed in support of this Viewpoint.
